# Assessing Resilience and Its Correlates among Residents of Fort McMurray during the COVID-19 Pandemic

**DOI:** 10.3390/ijerph20126064

**Published:** 2023-06-06

**Authors:** Nnamdi Nkire, Reham Shalaby, Gloria Obuobi-Donkor, Belinda Agyapong, Ejemai Eboreime, Vincent I. O. Agyapong

**Affiliations:** 1 Department of Psychiatry, Faculty of Medicine and Dentistry, University of Alberta, Edmonton, AB T6G 2R3, Canada; 2 Department of Psychiatry, Faculty of Medicine, Dalhousie University, Halifax, NS B3H 4R2, Canada

**Keywords:** anxiety, COVID-19, depression, pandemic, predictor, resilience, mental health

## Abstract

Background: The coronavirus disease of 2019 (COVID-19) pandemic has led to a global health crisis that has affected the psychological well-being of individuals across the world. The persistence of the pandemic and measures to curtail it have tested people’s ability to cope successfully and bounce back from the pandemic, otherwise referred to as resilience. The present study examined resilience levels among residents of Fort McMurray and identified the demographic, clinical and social factors associated with resilience. Methods: The study used a cross-sectional survey design and collected data from 186 participants using online questionnaires. The survey included questions assessing sociodemographic information, mental health history and COVID-19-related variables. The main study outcome was resilience measured using the six-item Brief Resilience Scale (BRS). The data from the survey were analyzed using chi-squared tests and binary logistic regression analyses in the Statistical Package for Social Sciences (SPSS), version 25. Results: The results showed that seven independent variables (age, history of depression, history of anxiety, willingness to receive mental health counselling, support from the government of Alberta and support from employer) were statistically significant within the context of the logistic regression model. A history of an anxiety disorder was demonstrated to best predict low resilience. Participants who had a history of anxiety disorder were five times more likely to show low resilience compared to those without such a history. Participants with a history of depression showed a three-fold likelihood of having low resilience in comparison to those who did not have a history of depression. Individuals who expressed a desire to receive mental health counselling had a four-times likelihood of having low resilience than those who did not express a desire to receive mental health counselling. The results also showed that younger participants were more prone to low resilience compared to older participants. Receiving support from the government and one’s employer is a protective factor. Conclusions: This study highlights the importance of examining resilience and its associated factors during a pandemic such as COVID-19. The results demonstrated that a history of anxiety disorder, depression and being younger were important predictors of low resilience. Responders who reported the desire to receive mental health counselling also reported expressing low resilience. These findings could be used to design and implement interventions aimed at improving the resilience of individuals affected by the COVID-19 pandemic.

## 1. Introduction

Life is full of challenges and adversities that can either strengthen or weaken individuals and societies. When faced with these challenges, individuals have the ability to overcome them, resulting in a sense of satisfaction and gratification. On the other hand, when these challenges or adversities are too much for an individual to handle, they can lead to negative consequences, such as physical and mental health disorders. Resilience, which is defined as the ability to successfully cope with significant changes, crises, risks, adversity and challenges, has become a popular term in modern psychology [1,2].

Resilience is a multidimensional psychological construct that has been defined in different ways, but it is generally considered as a positive adaptation in the face of adversity. While neuroscientists are interested in the concept, there are varying interpretations of what resilience entails [3]. For example, Wood and Bhatnagar (2015) believe that resilience is context-specific and dependent on the individual’s ability to adapt to the stressors in their environment [4]. Negative manifestations of resilience may result in psychological disorders such as anxiety disorder, major depressive disorder and post traumatic stress disorder (PTSD) [5]. On the other hand, resilience allows for societal and individual growth by providing a reservoir of strength and knowledge for future crises. It moderates distress that may arise from unexpected changes and outcomes, creating an environment that is necessary for confronting and resolving adversities without collapsing [6,7,8,9]. In recent times, the world has faced so many challenges which have tested societies’ resolve and individuals’ resilience; the economic crash of the late 2000s comes to mind, as well as political upheavals the world over. To add to these issues, the evolving environmental/climate change affecting the globe has imposed its burden on societies at large, with consequent stress upon the individual. All of these have had significant adverse effects on the economic fortunes of societies and individuals, creating feelings of panic and testing resilience. The most recent global phenomenon creating panic has been the coronavirus disease of 2019 (COVID-19) pandemic [10].

The COVID-19 pandemic has had a profound impact on communities worldwide, leading to numerous physical, social and mental health challenges [11,12,13,14,15]. Individuals were impacted directly or indirectly. There was a report of a notable increase in mental health difficulties during the COVID-19 pandemic; Nkire and Colleagues (2021) noted in their study that the one-week prevalence rate of generalized anxiety was 46.7%, and for major depressive disorder it was 41.4%. They further noted that the one-week prevalence for self-reported moderate to high stress was 84.9% [11]. These results were not dissimilar to findings in other studies examining reported depressive symptoms occurring in isolated individuals during the recent COVID-19 pandemic [16,17]. The pandemic caused widespread fear, uncertainty and economic hardship, highlighting the importance of psychological resilience in coping with the stressors associated with the pandemic. Like other cities across the world, Fort McMurray, located in the regional municipality of Wood Buffalo, Alberta, has been affected by the pandemic. Given its recent experiences with flooding and wildfires, as well as limited access to mental health services compared to other cities in Alberta [18,19], the residents of Fort McMurray have faced numerous challenges related to the pandemic, including job loss, isolation and mental health issues [10,11,14,16,20]. The study is the first of its type in Canada that we know of and specifically in a region such as Fort McMurry and adds to the data in this regard providing a reference for future research coming from Canada and Alberta. Consequently, this study aimed at evaluating resilience and its associated factors. The specific research goals were to (a) describe the levels of resilience in the residents of Fort McMurray in the course of the COVID-19 pandemic, and (b) to explore any relationships between resilience levels and the participants’ demographic, clinical and COVID-19-related characteristics.

## 2. Materials and Methods

### 2.1. Study Setting and Design

The University of Alberta Health Research Ethics Committee reviewed and approved this study. The study was set in Fort McMurray (FMM), Alberta. The 2016 Census of Population conducted by Statistics Canada put the population of Fort McMurray at 66,573 persons living in 23,937 of its 28,567 total private dwellings [21,22]. A cross-sectional design was adopted where survey questionnaires were sent out between 24 April and 2 June 2021 to residents of FMM. The questionnaires were administered via Research Electronic Data Capture (REDcap) [23]. The random distribution of questionnaires utilized emails via community, government, school and occupational platforms. The inclusion criteria included individuals aged 18 years and above who were living in FMM during the COVID-19 pandemic. Those residing in FMM for less than a year and temporary residents were excluded. Data collected included socio-demographic, resilience, clinical, COVID-related questions and level of support from various governments. Details about the survey were provided to the incepted cohort. Consent was implied via the completion of the survey.

### 2.2. Sample Size Estimation

The 2018 Census determined that approximately 111,687 residents inhabited FMM at the time of the 2018 Census. From this sample size, utilizing a confidence interval of 95% with a +/−5% margin of error, we estimated that the sample size required for prevalence estimates of likely low resilience for mental disorders was 383.

### 2.3. Outcome Measure

The Brief Resilience Scale (BRS) was utilized to assess resilience in this survey. The BRS is a 6-item scale used to assess the perceived ability to bounce back or recover from stress. At its core, it is a unitary construct of resilience, including both positively and negatively worded items [24]. Each item is scored on a graded scale from 1 to 5. Scoring is achieved by adding the value (1–5) of responses for all six items; the total score obtained is divided by the total number of questions, i.e., 6 for the final score. Low resilience lies between scores of 1.00 and 2.99 and high resilience is when scores lie between 4.31 and 5.00; values between high and low resilience scores are considered to be normal resilience, i.e., 3.00 to 4.30. The BRS has good internal consistency, with a range of 0.80–0.90 Cronbach’s alpha [24]. For the purpose of analyses, normal and high resilience categories were merged into one category. Using this merger, we ended up with two main categories: the low resilience category and the normal-to-high resilience category.

The BRS represented the main study outcome. Other variables studied included demography, medication history, mental health of participants and willingness to receive counselling. Exposure to COVID-19 pandemic news was also explored. Finally, the levels of support provided by family, friends, employers and the government during the pandemic were explored.

### 2.4. Statistical Analysis

Data analysis was carried out using Statistical Package for Social Sciences, version 25 (IBM Corp 2011, New York, NY, USA) [25]. Descriptive statistics were used to present the variables examined, including clinical, demographic and COVID-19-related variables. Chi-squared analysis was conducted to explore the relationship between variables and resilience levels, i.e., low and normal to high resilience. In cases where the expected number counts were less than 5 in a cell, we utilized Fisher’s exact test instead. Binary logistic regression analysis was carried out to ascertain predictors of low resilience, with significance levels set at *p* ≤ 0.05 or near significance (0.1 > *p* > 0.05). Odds ratios (ORs) and confidence intervals were reported. We deployed correlational analyses (Spearman’s correlation coefficient of 0.7 to 1.0 or −0.7 to −1.0) so as to exclude any strong intercorrelations.

## 3. Results

Of the 247 persons who accessed our survey link, 186 actually completed the survey. This gave a survey completion rate of 74.7%. Table 1 displays information on clinical, demographic and COVID-19-related data. As seen from the table, 93% of respondents were aged 26 years or older and most were female (86%). Out of this number, 94% of the respondents were divorced, separated or widowed, 87% were married, partnered or cohabiting and 75% were single. A total of 94% of the participants were employed. Of those employed, half (50%) worked with the school boards. Regarding clinical variables, 31% and 42% had a history of depression and anxiety, respectively. In addition, 48.4% reported having no history of mental health diagnosis, 36% were on psychotropic medication, 39% had received mental health counselling in the past year and more than half were willing to receive mental health counselling (53%). In terms of COVID-19-related variables, 92% of participants were fearful of contracting coronavirus, 97% were fearful about their close friends or family members contracting the virus and 72% reported that their close friends and family actually contracted the virus. More than half of the participants had to self-quarantine or isolate themselves due to COVID-19 symptoms, recent travel or coming into contact with an infected individual. During the pandemic, the majority (58%) read newspaper and Internet reports related to the pandemic daily. About 44% on a daily basis viewed television content related to sick and dead people who had contracted the COVID-19 virus. Approximately 88% remained in their jobs despite the virus. We noted that while 44% received good support from family and friends, the number was almost the same as those who received absolute support from their employer (45%), and less than those who reported no support from the governments of Canada and Alberta (54% and 63%, respectively). Regarding the level of resilience among the study respondents, 64 (37.4) expressed low resilience.

Table 2 represents the univariate analysis included in the study. It highlights the relationships between resilience and variable factors such as clinical, demographic and COVID-19-related factors. Statistical significance was demonstrated using the chi-square/Fisher’s exact test in examining associations between resilience and a host of variables including age, employment history, history of depression, history of anxiety, history of any mental health diagnosis from a healthcare professional, receiving antidepressant medications, receiving mental health counselling in the past, willingness to receive mental health counselling, family members contracting the virus and receiving sufficient support from family and friends, the government of Canada, the government of Alberta and one’s employer since the COVID-19 pandemic was declared.

The multivariate logistic regression model (Table 3) shows the association between low resilience and each variable in the cohort after controlling for other variables in the model. There were nine variables in the model which achieved significance with *p* values <0.05. These were then computed in a logistic regression model. The nine predictors were statistically significant and accounted for approximately 36.2% (Cox and Snell R^2^) to 49.2% (Nagelkerke R^2^) of the variance seen, and correctly classified about 78.9% of all cases. It should be noted that certain variables—including history of mental health diagnosis, receiving antidepressants and receiving support from the government of Canada—were excluded from the model because they demonstrated a high correlation with other variables (r_s_ > 0.7). Table 3 shows that the main contributors to statistical significance in the model were age, a history of depression, a history of anxiety, willingness to receive mental health counselling, support from the government of Alberta and support from one’s employer. The main predictor of low resilience was having a history of an anxiety disorder (Wald = 8.001) (OR = 0.203; 95% CI 0.067–0.613). This suggests that participants with a history of anxiety disorder demonstrated a five-fold likelihood of having low resilience in comparison to those who had no history of anxiety. Participants who had a history of depression had a three-fold likelihood of showing low resilience compared to participants without a history of depressive disorder (OR = 0.313; 95% CI: 0.109–0.902). As well, individuals who “would like to receive mental health counselling” were four times more likely to exhibit low resilience compared to those who would not like to receive mental health counselling. Similarly, age made a significant contribution to the model. Participants 25 years old or younger were more prone to experience low resilience. Those aged 40 years or older were 18.5 times less likely to show low resilience compared to those individuals who were aged 25 years and younger (OR = 0.054; 95% CI: 0.006–0.470). Those aged 25 years and younger had a 17-fold-more likelihood of expressing low resilience than individuals who were between the ages of 26 and 40 years old (OR = 0.59; 95% CI: 0.006–0.546). Individuals who received limited support from the government of Alberta following the declaration of the COVID-19 pandemic were less likely to demonstrate low resilience in comparison to those who received absolute support from the same government during the same time (OR = 19.575; 95% CI: 1.606–238.6664). In a similar manner, individuals who received no support from their employer during the COVID-19 pandemic were eight times less likely to express low resilience than those who received absolute support from their employers during the same period.

## 4. Discussion

The present study examined the construct of resilience and the factors affecting it amongst those who resided in Fort McMurray during the COVID-19 pandemic. It provides valuable insight into how the pandemic affected the residents of Fort McMurray, who, it must be noted, have experienced multiple traumas in recent times. The results of the study showed that several factors were associated with low resilience among the participants. The strongest predictor of low resilience was found to be a history of anxiety disorder. Individuals who reported having a history of anxiety were five times more likely to report low resilience in comparison to those without a similar history. As well, having a history of depressive disorder, being 25 years or younger and expressing a willingness to receive mental health counselling were shown to predict low resilience in a statistically significant manner. The relationship observed in this study of an opposite relationship between resilience and anxiety is similar to that reported in a Chinese cohort in 2020 by Zhang et al., who suggested that resilience and reported anxiety/depression had an inverse relationship, with the levels of demonstrated resilience rising as the levels of reported anxiety and depression depreciated, and that resilience can be protective against these conditions in COVID-19 patients with mild symptoms [26]. As well, in a cohort of Israeli physicians studied during the COVID-19 pandemic, Mosheva and colleagues (2020) found an inverse relationship between resilience and anxiety [27]. A study by Killgore and colleagues (2020) in the US reported similar findings for depression and anxiety in relation to resilience using different measuring tools [28]. The available literature report contradicting correlations between anxiety and depression and level of resilience. For example, Sampogna and colleagues (2021) found in their study that the level of depression and anxiety had no influence on the levels of resilience [29] among their sample in Italy [29].

As previously stated, our cohort had been exposed to other traumatic events in the preceding years. It is likely that these events influenced the ability of this cohort to deal with the pandemic. It has been suggested that positive emotions and emotional flexibility have a relationship with resiliency levels [30,31,32], and that this combination improved one’s adaptability to and ability to cope with the pandemic. A recent study by Adu and colleagues (2022) examining resilience five years after the wildfires in FMM reported that PTSD was correlated to resilience in this population and was a statistically significant predictor of low resilience [33]. This finding is in keeping with our report on the relationship between resilience and anxiety in this cohort during the COVID-19 pandemic.

The relationship between resiliency levels and support from the government and one’s employer during the COVID-19 pandemic, as demonstrated in this study, suggests that the level of support from the community and employers played an important role in determining resilience during the COVID-19 pandemic. Similarly, a study by Sampogna et al. (2021) reported that only practical support lowered stress levels, while emotional support improved resilience levels during the pandemic [29].

These findings highlight the need for targeted interventions that address the mental health needs of communities affected by COVID-19 and provide adequate support to those in need.

This study further showed that age was positively correlated with resilience, with those >40 years old likely to present with higher resilience. This is consistent with prior studies, which demonstrate that as people age, they become more resilient [34]. This may be related to resilience built secondary to previous exposures to adverse life events and learning to cope with these. Higher resilience may be protective for older adults, allowing them to compensate for declines in functional capacity and physical health, resulting in better health outcomes and less depression [35]. However, amongst older people, resilience is shown to be worse amongst those with chronic conditions and physical limitations. These individuals are used to having more support from family, friends and health services. With isolation and quarantine measures in the early part of the COVID-19 pandemic, these individuals were more likely to have been adversely impacted and to decompensate mentally. In pandemics and periods of isolation and quarantine, these subgroups of older personnel may benefit from the use of the Internet and mobile technology [36]. The COVID-19-related questions did not have any effect on the study population. This may be explained by respondents developing resilience from previous experience. Additionally, the questionnaire was also administered at a time when the pandemic caused relatively few restrictions and consequences on daily life compared to a year earlier, which may have affected their response to resilience.

The study of resilience during times of crisis is essential for understanding the factors that help individuals and communities to cope with adversity. The findings of the present study may aid in the development of targeted interventions aimed at promoting resilience and reducing the adverse effects of the pandemic on health, especially on mental health. Additionally, findings from this study may provide a window into the needs of communities affected by COVID-19 and inform future efforts to support those in need. By gaining a better understanding of the factors associated with resilience, individuals and communities can be better equipped to handle adversity, allowing them to bounce back from negative experiences and grow stronger as a result. Ultimately, by promoting resilience, individuals and communities can be better prepared to handle the challenges that life may throw their way, leading to greater overall health and well-being.

A limitation of this study is our inability to estimate the actual response rate in this survey, as it was impossible to determine the number of individuals who received the invitation from the community partners to complete the survey. However, this online survey achieved a reasonably high survey completion rate relative to the individuals who accessed or clicked on the survey link. A lower response rate poses a challenge in ensuring the representativeness of the sampled population [37]. The high survey completion rate may be due to the high number of young people and female participants who accessed this survey link; previous research has shown that young people and females demonstrated a greater likelihood for completing surveys in comparison to their male counterparts [38]. The cross-sectional design of this study poses a limitation, as it only provides a snapshot of the participants’ resilience levels and its correlates at a single point in time and may not accurately capture changes in resilience levels over time. Additionally, as the survey was administered via email and distributed via various platforms, the results may have been influenced by self-selection bias, where only individuals with strong opinions on the topic may have chosen to participate. There is also the potential for social desirability bias, where participants may have provided socially acceptable answers rather than their true experiences and feelings. This is however unlikely due to the online and anonymous nature of the survey. Furthermore, Bonanno (2021) explained that a self-administered scale to assess resilience can lead to biases [39]. The measures used in this study were self-reported, which may not accurately reflect the true experiences of the participants. The use of self-reported measures may also have led to recall bias, where participants may not accurately remember their experiences and perceptions. Finally, generalizability is adversely impacted by the limited sample size of the population. As such, more studies with larger sample sizes are required in furtherance of the objectives of the present study.

## 5. Conclusions

This study shines a light on the factors associated with resilience during a pandemic such as COVID-19. It can form a template upon which interventions are based to treat depression and anxiety by strengthening resilience and improving available supports and services to those in need, including young adults and those who are not receiving support from their employers. These findings are within the reported range of the published literature and broadly in accordance with the mental health and COVID-related literature.

The results of this study highlight the need for continued research into resilience and its associated factors during the COVID-19 pandemic, so as to inform effective interventions for those in need, for this and for future pandemics.

## Figures and Tables

**Table 1:** Demographic characteristics of the sample.

Variables	Married/Partnered/ Cohabiting *n* (%)	Divorced/ Separated/Widowed *n* (%)	Single *n* (%)	Total *n* (%)
**Gender**				
Male	17 (12.9)	1 (5.6)	9 (25.0)	27 (14.5)
Female	115 (87.1)	17 (94.4)	27 (75.0)	159 (85.5)
**Age (years)**				
≤25 y	3 (2.3)	0 (0.0)	10 (27.8)	13 (7.0)
26–40 y	59 (44.7)	2 (11.1)	14 (38.9)	75 (40.3)
>40 y	70 (53.0)	16 (88.9)	12 (33.3)	98 (52.7)
**Employment status**				
Employed	125 (94.7)	18 (100.0)	32 (88.9)	175 (94.1)
Unemployed	7 (5.3)	0 (0.0)	4 (11.1)	11 (5.9)
**Employment place**				
School boards	65 (52.4)	6 (33.3)	16 (50.0)	87 (50.0)
Healthcare industry	9 (7.3)	0 (0.0)	1 (3.1)	10 (5.7)
Keyano College	14 (11.3)	4 (22.2)	2 (6.3)	20 (11.5)
Oil sands industry	8 (6.5)	1 (5.6)	4 (12.5)	13 (7.5)
Municipal or government agency	11 (8.9)	1 (5.6)	1 (3.1)	13 (7.5)
Other	17 (13.7)	6 (33.3)	8 (25.0)	31 (17.8)
**History of mental health diagnosis**				
Depression	37 (28.0)	9 (50.0)	12 (33.3)	58 (31.2)
Bipolar disorder	3 (2.3)	1 (5.6)	2 (5.6)	6 (3.2)
Anxiety	54 (40.9)	9 (50.0)	15 (41.7)	78 (41.9)
Schizophrenia	0 (0.0)	0 (0.0)	0 (0.0)	0 (0.0)
Personality disorder	0 (0.0)	1 (5.6)	1 (2.8)	2 (1.1)
Other	12 (9.1)	0 (0.0)	5 (13.9)	17 (9.1)
No mental health diagnosis	69 (52.3)	5 (27.8)	16 (44.4)	90 (48.4)
**History of psychotropic medications**				
On psychotropic medication	40 (30.3)	9 (50.0)	17 (47.2)	66 (35.5)
**History of MH counselling**	48 (36.4)	8 (44.4)	16 (44.4)	72 (38.7)
**Desire to receive MH counselling**	66 (50.0)	11 (61.1)	21 (58.3)	98 (52.7)
**Fear of contracting coronavirus**	113 (89.7)	17 (100.0)	30 (96.8)	160 (92.0)
**Fearful about close relations contracting coronavirus**	121 (96.0)	17 (100.0)	30 (96.8)	168 (96.6)
**Close relation sick from coronavirus disease**	94 (75.8)	10 (58.8)	20 (64.5)	124 (72.1)
**Self-isolation/quarantine due to COVID-19 symptoms**				
Yes	77 (61.6)	11 (64.7)	16 (51.6)	104 (60.1)
No	48 (38.4)	6 (35.3)	15 (48.4)	69 (39.9)
**Frequency watching television content of sick and dead people caused by coronavirus**				
Daily	58 (46.0)	10 (58.8)	9 (29.0)	77 (44.3)
Less than daily	53 (42.1)	7 (41.2)	14 (45.2)	74 (42.5)
Not at all	15 (11.9)	0 (0.0)	8 (25.8)	23 (13.2)
**Frequency reading newspaper and Internet articles**				
Daily	72 (57.1)	11 (64.7)	18 (58.1)	101 (58.0)
Less than daily	50 (39.7)	6 (35.3)	13 (41.9)	69 (39.7)
Not at all	4 (3.2)	0 (0.0)	0 (0.0)	4 (2.3)
**Lose job due to the COVID-19 pandemic**				
Yes	14 (11.1)	4 (23.5)	3 (9.7)	21 (12.1)
No	112 (88.9)	13 (76.5)	28 (90.3)	153 (87.9)
**Support from family and friends**				
Absolute support	56 (44.8)	6 (35.3)	14 (45.2)	76 (43.9)
Some support	38 (30.4)	7 (41.2)	10 (32.3)	55 (31.8)
Limited support	18 (14.4)	3 (17.6)	5 (16.1)	26 (15.0)
No support	13 (10.4)	1 (5.9)	2 (6.5)	16 (9.2)
**Support from the government of Canada since the COVID-19 pandemic was declared**				
Absolute support	18 (14.6)	0 (0.0)	4 (12.9)	22 (13.0)
Some support	20 (16.3)	4 (26.7)	3 (9.7)	27 (16.0)
Limited support	21 (17.1)	3 (20.0)	4 (12.9)	28 (16.6)
No support	64 (52.0)	8 (53.3)	20 (64.5)	92 (54.4)
**Support from the government of Alberta**				
Absolute support	12 (9.8)	0 (0.0)	4 (12.9)	16 (9.4)
Some support	20 (16.3)	3 (18.8)	2 (6.5)	25 (14.7)
Limited support	16 (13.0)	1 (6.3)	5 (16.1)	22 (12.9)
No support	75 (61.0)	12 (75.0)	20 (64.5)	107 (62.9)
**Support from employer**				
Absolute support	56 (45.2)	10 (58.8)	12 (38.7)	78 (45.3)
Some support	34 (27.4)	3 (17.6)	9 (29.0)	46 (26.7)
Limited support	17 (13.7)	4 (23.5)	3 (9.7)	24 (14.0)
No support	17 (13.7)	0 (0.0)	7 (22.6)	24 (14.0)
Resilience				
High-to-normal resilience	79 (63.7)	11 (68.8)	17 (54.8)	107 (62.6)
Low resilience	45 (36.3)	5 (31.3)	14 (45.2)	64 (37.4)

**Table 2:** Chi-square test of association between variables and likely low resilience.

Variables	Low Resilience	High to Normal Resilience	Chi-Square/ Fisher Exact	*p*-Value
Demographic characteristics				
**Gender**			1.841	0.255
Male	6 (25.0)	18 (75.0)		
Female	58 (39.5)	89 (60.5)		
**Age categories**			8.098	0.015
≤25	7 (77.8%)	2 (22.2%)		
26–40	28 (40.6%)	41 (59.4%)		
>40	29 (31.2%)	64 (68.8%)		
**Employment status**			8.220	0.006
Employed	56 (34.8%)	105 (65.2%)		
Unemployed	8 (80.0%)	2 (20.0%)		
**Place of employment**			3.454 *	0.642
School boards	27 (34.6%)	51 (65.4%)		
Healthcare industry	3 (33.3%)	6 (66.7%)		
Keyano College	6 (30.0%)	14 (70.0%)		
Oil sands industry	5 (41.7%)	7 (58.3%)		
Municipal or government agency	2 (16.7%)	10 (83.3%)		
Other	13 (44.8%)	16 (55.2%)		
**Housing status**			1.474	0.247
Own home	54 (39.7%)	82 (60.3%)		
Renting	10 (28.6%)	25 (71.4%)		
Clinical characteristics				
**History of depression**			21.976	0.000
Yes	34 (63.0%)	20 (37.0%)		
No	30 (25.6%)	87 (74.4%)		
**History of bipolar disorder from a health professional**			0.044	1.000
Yes	2 (33.3%)	4 (66.7%)		
No	62 (37.6%)	103 (62.4%)		
**History of anxiety from a health professional**			20.229	0.000
Yes	41 (56.9%)	31 (43.1%)		
No	23 (23.2%)	76 (76.8%)		
**History of personality disorder**			0.602	1.000
Yes	1 (100%)	0 (0.0%)		
No	106 (62.4%)	64 (37.6%)		
**History of any mental health diagnosis**			16.112	0.000
Yes	43 (48.3%)	46 (51.7%)		
No	64 (78.0%)	18 (22.0%)		
**Received antidepressants in the past**			11.076	0.001
Yes	30 (55.6%)	24 (44.4%)		
No	34 (29.1%)	83 (70.9%)		
**History of antipsychotic medication**			0.276	0.631
Yes	2 (50.0%)	2 (50.0%)		
No	105 (62.9%)	62 (37.1%)		
**Respondents received MH counselling in the past year**			7.971	0.006
Yes	33 (50.8%)	32 (49.2%)		
No	31 (29.2%)	75 (70.8%)		
**Respondents would like to receive MH counselling**			24.630	0.000
Yes	49 (55.1%)	40 (44.9%)		
No	15 (18.3%)	67 (81.7%)		
COVID-19-related characteristics				
**Fearful of contracting coronavirus**			1.666	0.158
Yes	61 (38.9)	96 (61.1)		
No	3 (21.4)	11 (78.6)		
**Fearful of their close relations contracting coronavirus**			3.719	0.085 *
Yes	64 (38.8)	101 (61.2)		
No	0 (0.0)	6 (100.0)		
**Friends or family members sick from coronavirus disease**			0.084	0.861
Yes	45 (37.2)	76 (62.8)		
No	19 (39.6)	29 (60.4)		
**Self-isolation/quarantine due to COVID-19 symptoms**			0.267	0.631
Yes	40 (39.2)	62 (60.8)		
No	24 (35.3)	44 (64.7)		
**Frequency watching television content of sick people/people with COVID-19**			1.074	0.606
Daily	29 (38.7)	46 (61.3)		
Less than daily	25 (33.8)	49 (66.2)		
Not at all	10 (45.5)	12 (54.5)		
**Frequency reading newspapers or articles related to the pandemic**			3.322	0.184 *
Daily	39 (39.4)	60 (60.6)		
Less than daily	22 (32.4)	46 (67.6)		
Not at all	3 (75.0)	1 (25.0)		
**Lost your job due to COVID-19**			2.286	0.152
Yes	11 (52.4)	10 (47.6)		
No	53 (35.3)	97 (64.7)		
**Support from family and friends**			9.921	0.018
Absolute support	20 (26.7)	55 (73.3)		
Some support	20 (37.0)	34 (63.0)		
Limited support	15 (57.7)	11 (42.3)		
No support	8 (53.3)	7 (46.7)		
**Support from the government of Canada**			8.854	0.030
Absolute support	2 (9.1)	20 (90.9)		
Some support	11 (42.3)	15 (57.7)		
Limited support	12 (42.9)	16 (57.1)		
No support	38 (41.8)	53 (58.2)		
**Support from the government of Alberta**			12.078	0.005 *
Absolute support	1 (6.3)	15 (93.8)		
Some support	7 (29.2)	17 (70.8)		
Limited support	13 (59.1)	9 (40.9)		
No support	43 (40.6)	63 (59.4)		
**Support from employer**			25.798	0.000
Absolute support	19 (24.4)	59 (75.6)		
Some support	18 (39.1)	28 (60.9)		
Limited support	8 (34.8)	15 (65.2)		
No support	19 (82.6)	4 (17.4)		

* Fisher’s exact test. MH—mental health.

**Table 3:** Logistic regression model for respondents’ likelihood to present with low resilience during the pandemic.

Variables		Coefficient	Standard Error	Wald Statistic	*p*-Value	Odds Ratio	95% Confidence Interval	
Age (years)							**Lower**	**Upper**
	≤25			7.007	0.030			
	26–40	−2.832	1.136	6.214	0.013	0.059	0.006	0.546
	≥40	−2.922	1.106	6.981	0.008	0.054	0.006	0.470
Employment status		−0.105	1.104	0.009	0.924	0.901	0.103	7.843
Clinical history	Depression	−1.161	0.539	4.630	0.031	0.313	0.109	0.902
	Anxiety	−1.595	0.564	8.001	0.005	0.203	0.067	0.613
	Not on any medication for mental health concerns	0.561	0.581	0.934	0.334	1.752	0.562	5.467
	Received mental health counselling in the past year	0.699	0.587	1.417	0.234	2.011	0.636	6.357
	Would you like to receive mental health counselling?	−1.326	0.534	6.172	0.013	0.265	0.093	0.756
Received support from family and friends since the COVID-19 pandemic was declared	Absolute			1.086	0.781			
	Some support	−0.305	0.528	0.333	0.564	0.737	0.262	2.076
	Limited support	0.231	0.700	0.109	0.741	1.260	0.319	4.973
	No support	0.406	0.823	0.243	0.622	1.501	0.299	7.530
Received support from the government of Alberta since the COVID-19 pandemic was declared	Absolute support			6.282	0.099			
	Some support	2.032	1.289	2.487	0.115	7.630	0.610	95.369
	Limited support	2.974	1.276	5.434	0.020	19.575	1.606	238.664
	No support	1.908	1.186	2.590	0.108	6.741	0.660	68.852
Received support from employer since the COVID-19 pandemic was declared	Absolute support			7.976	0.047			
	Some support	0.449	0.508	0.783	0.376	1.567	0.579	4.239
	Limited support	−0.182	0.664	0.075	0.784	0.834	0.227	3.060
	No support	2.100	0.786	7.135	0.008	8.165	1.749	38.120

## Data Availability

Data will be made available upon reasonable request to the corresponding author.
